# Fucoxanthin Enhances the Level of Reduced Glutathione via the Nrf2-Mediated Pathway in Human Keratinocytes

**DOI:** 10.3390/md12074214

**Published:** 2014-07-15

**Authors:** Jian Zheng, Mei Jing Piao, Ki Cheon Kim, Cheng Wen Yao, Ji Won Cha, Jin Won Hyun

**Affiliations:** School of Medicine and Institute for Nuclear Science and Technology, Jeju National University, Jeju 690-756, Korea; E-Mails: zhengjian0317@hotmail.com (J.Z.); mjpiao@hanmail.net (M.J.P.); svv771@hotmail.com (K.C.K.); vane1989923@hotmail.com (C.W.Y.); cjw102700@hanmail.net (J.W.C.)

**Keywords:** fucoxanthin, NF-E2-related factor 2, oxidative stress, cytoprotection, PI3K/Akt, GCLC, GSS, GSH

## Abstract

Fucoxanthin, a natural carotenoid, is abundant in seaweed with antioxidant properties. This study investigated the role of fucoxanthin in the induction of antioxidant enzymes involved in the synthesis of reduced glutathione (GSH), synthesized by glutamate-cysteine ligase catalytic subunit (GCLC) and glutathione synthetase (GSS), via Akt/nuclear factor-erythroid 2-related (Nrf2) pathway in human keratinocytes (HaCaT) and elucidated the underlying mechanism. Fucoxanthin treatment increased the mRNA and protein levels of GCLC and GSS in HaCaT cells. In addition, fucoxanthin treatment promoted the nuclear translocation and phosphorylation of Nrf2, a transcription factor for the genes encoding GCLC and GSS. Chromatin immune-precipitation and luciferase reporter gene assays revealed that fucoxanthin treatment increased the binding of Nrf2 to the antioxidant response element (ARE) sequence and transcriptional activity of Nrf2. Fucoxanthin treatment increased phosphorylation of Akt (active form), an up-regulator of Nrf2 and exposure to LY294002, a phosphoinositide 3-kinase (PI3K)/Akt inhibitor, suppressed the fucoxanthin-induced activation of Akt, Nrf2, resulting in decreased GCLC and GSS expression. In accordance with the effects on GCLC and GSS expression, fucoxanthin induced the level of GSH. In addition, fucoxanthin treatment recovered the level of GSH reduced by ultraviolet B irradiation. Taken together, these findings suggest that fucoxanthin treatment augments cellular antioxidant defense by inducing Nrf2-driven expression of enzymes involved in GSH synthesis via PI3K/Akt signaling.

## 1. Introduction

Oxidative stress is the most common cause of skin aging and can be effectively eliminated by the organism itself, pharmacological agents, and natural antioxidants. There are two theoretical methods to deal with these harmful stimuli, namely, early and delayed responses. Early responses rapidly remove reactive oxygen species (ROS) and free radicals via chemical reactions soon after their generation [1]. By contrast, delayed responses involve the expression of genes encoding antioxidant enzymes and proteins to reduce the generation of noxious substances [2]. Nuclear factor-erythroid 2-related factor (Nrf2) is often the central signaling switch that modulates the activation of phase II bio-transferase/antioxidant enzymes, which include glutamate-cysteine ligase catalytic subunit (GCLC) and glutathione synthetase (GSS) [3,4]. As an extremely important antioxidant, GSH, which is synthesized by GCLC and GSS [5,6], not only scavenges free radicals [7], but also maintains the redox-sensitive active sites of many enzymes from an oxidized form to a reduced form [8]. Therefore, the correct balance between reduced GSH and oxidized GSH is required for cellular homeostasis [9].

Genes that encode antioxidant enzymes, such as GCLC and GSS, contain an antioxidant responsive element (ARE) in their promoter region [3]. Transduction of the ARE sequence-containing genes encoding GCLC and GSS mainly occurs via activation of Nrf2 protein [10]. Nrf2 is a transcription factor that detects variation in oxidative stress within cells [11] and induces the transcription of its target genes by binding to the ARE in their promoters. The target genes of Nrf2 include many antioxidant and phase II detoxifying genes [12], including those encoding GCLC and GSS. The synthesis of GSH catalyzed by GCLC and GSS via up-regulation of Nrf2 is associated with protection of cells against oxidative stress [13].

Fucoxanthin is a major carotenoid found in edible brown seaweeds [14] and contains several functional groups, including an unusual allenic bond, a conjugated carbonyl group, and an acetyl group [15]. Fucoxanthin has antioxidant, anti-cancer, anti-obesity, anti-diabetic, and anti-photoaging activities [16,17,18,19,20]. We previously reported that fucoxanthin reduced levels of ROS, inhibited DNA damage, restored mitochondrial membrane potential, and suppressed apoptosis [21]. In the present study, we examined whether fucoxanthin could increase the level of GSH by inducing GCLC and GSS expression via the Akt/Nrf2 pathway.

## 2. Results

### 2.1. Fucoxanthin Increases Expression of GCLC and GSS at the mRNA and Protein Levels

**Figure 1:**
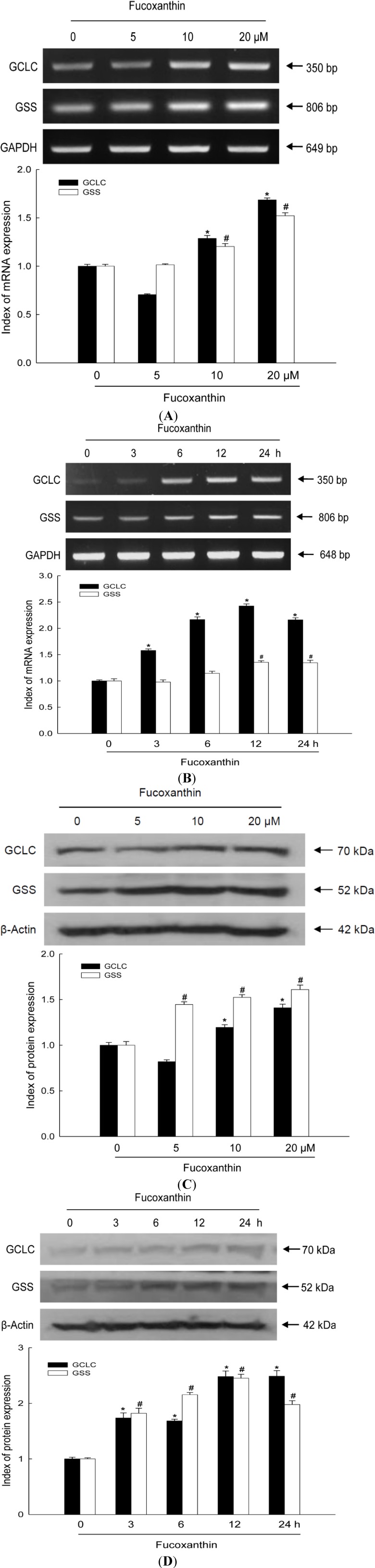
Effects of fucoxanthin treatment on the mRNA and protein expression of glutamate-cysteine ligase catalytic subunit (GCLC) and glutathione synthetase (GSS) in HaCaT cells. Cells were incubated with various concentrations of fucoxanthin (0–20 μM) for various amounts of time (0–24 h). The mRNA levels of GCLC and GSS were detected by reverse transcription-PCR (RT-PCR) following treatment (**A**) with variousconcentrations of fucoxanthin for 12 h; and (**B**) with 20 μM fucoxanthin for various amounts of time. The protein levels of GCLC and GSS were detected by Western blotting following treatment (**C**) with various concentrations of fucoxanthin for 12 h; and (**D**) with 20 μM fucoxanthin for various amounts of time. * and **^#^** indicates significantly different from control of GCLC and GSS, respectively (*p* < 0.05).

HaCaT cells were treated with various concentrations of fucoxanthin for 12 h. The mRNA levels of GCLC and GSS in fucoxanthin-treated cells increased, demonstrating that 20 μM of fucoxanthin caused the maximal induction both in GCLC and GSS expression (Figure 1A). Next, HaCaT cells were treated with 20 μM fucoxanthin for various amounts of time. The mRNA levels of GCLC and GSS were highest at 12 h of treatment (Figure 1B). Furthermore, when HaCaT cells were treated with various concentrations of fucoxanthin for 12 h, the protein levels of GCLC and GSS increased, exhibiting that 20 μM of fucoxanthin induced maximal in GCLC and GSS expression (Figure 1C). Treatment with 20 μM of fucoxanthin for 12 h markedly increased the protein levels of GCLC and GSS (Figure 1D). These results indicate that fucoxanthin treatment increases expression of GCLC and GSS at both the mRNA and protein levels.

### 2.2. Fucoxanthin Induces Activation of Nrf2 and Enhances Binding of Nrf2 to the ARE in the Promoters of the GCLC and GSS Genes

**Figure 2:**
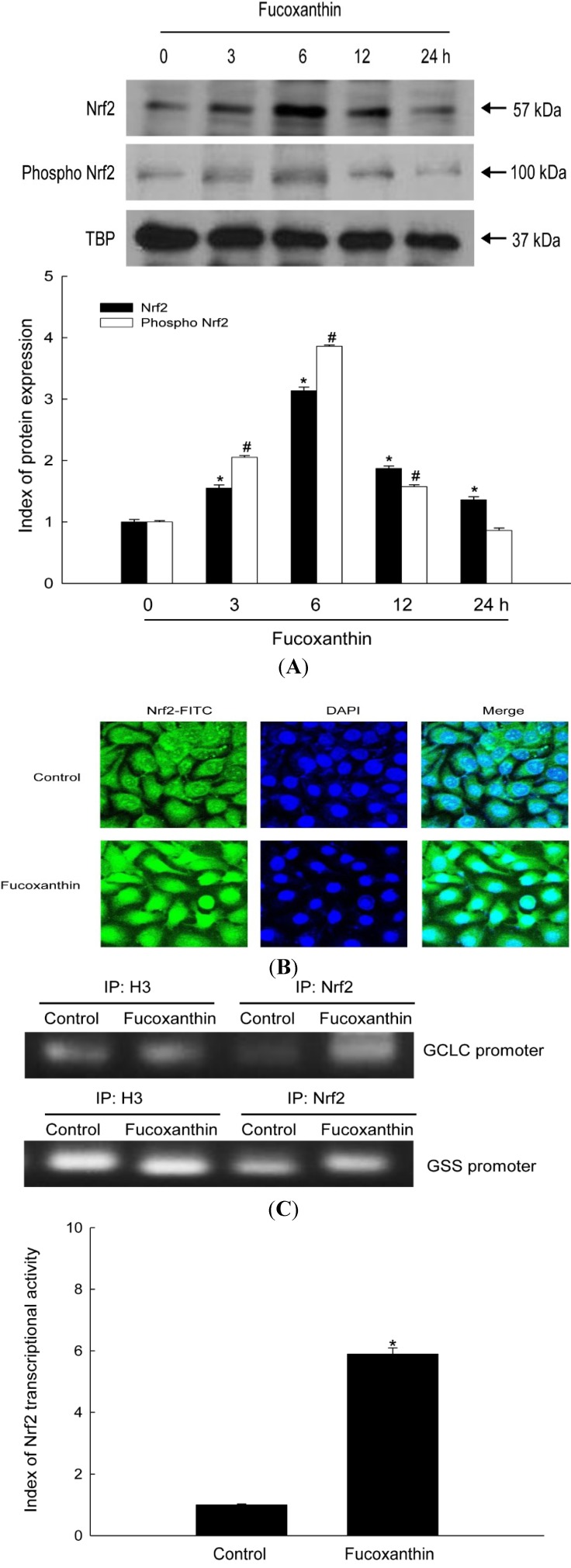
Effects of fucoxanthin treatment on the expression, nuclear translocation, and antioxidant response element (ARE) sequence-binding activity of Nrf2. (**A**) Nuclear extracts were prepared from HaCaT cells following treatment with 20 μM fucoxanthin for the indicated amount of time. Western blotting of the nuclear lysates was performed using Nrf2 and phospho Nrf2 antibodies. * and **^#^** indicates significantly different from Nrf2 and phospho Nrf2 of control, respectively (*p* < 0.05); (**B**) An anti-Nrf2 antibody and a FITC-conjugated secondary antibody were used to detect Nrf2 localization (green) by using confocal microscopy.DAPI staining indicates the locations of nuclei (blue). The merged images show the nuclear localization of Nrf2 protein; (**C**) Nuclear extracts were prepared from HaCaT cells treated with 20 μM fucoxanthin for 6 h. A ChIP assay was performed to assess binding of Nrf2 to the ARE in the promoters of the genes encoding GCLC and GSS; (**D**) Transcriptional activity of Nrf2 in HaCaT cells following treatment with 20 μM fucoxanthin for 6 h was assessed by using luciferase reporter assay. * Significantly different from control cells.

The genes encoding GCLC and GSS have an ARE sequence in their promoter regions. Nrf2 is an important transcription factor that regulates ARE-driven expression of these genes [22]. We examined whether fucoxanthin treatment activated Nrf2, resulting in the up-regulation of these enzymes. Fucoxanthin treatment increased protein levels of Nrf2 and phospho Nrf2 (active form) (Figure 2A), and resulted in the translocation of Nrf2 protein from the cytosol into the nucleus (Figure 2B). Moreover, chromatin immune-precipitation (ChIP) analysis revealed that binding of Nrf2 to the ARE in the promoters of the genes encoding GCLC and GSS was markedly increased in fucoxanthin-treated cells, as determined by comparison to binding of histone H3 as the internal control (Figure 2C). To verify the functional relevance of Nrf2 binding to the ARE sequence of these two genes, a construct was used that contained a promoter containing an ARE sequence (bearing the consensus Nrf2-binding site) linked to a luciferase reporter gene. Fucoxanthin treatment increased the transcriptional activity of Nrf2 (Figure 2D). These results suggest that Nrf2 mediates fucoxanthin-induced transcription of GCLC and GSS.

### 2.3. Fucoxanthin Involves Nrf2-Driven GCLC and GSS via Phosphorylation of Akt

**Figure 3:**
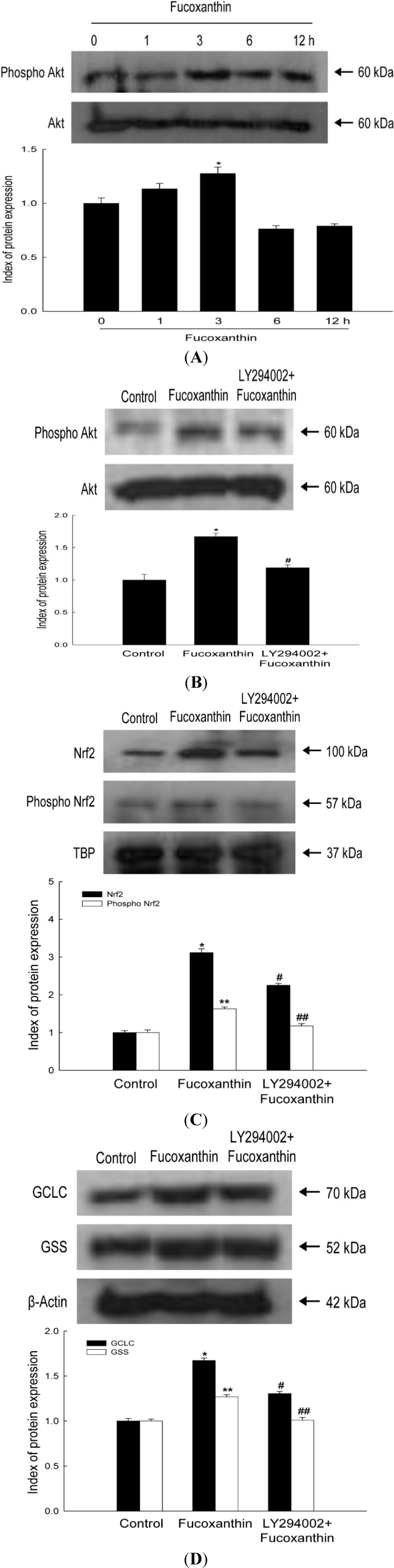
Effects of fucoxanthin treatment on Akt and its related protein. (**A**) Cells were incubated with 20 μM fucoxanthin for various amounts of time (0–12 h). Cell lysates were prepared and Western blotting was performed with anti-Akt and anti-phospho Akt antibodies. * indicates significantly different from control cells (*p* < 0.05); After treatment with LY294002, cell lysates were subjected to electrophoresis (**B**) with anti-Akt, anti-phospho Akt. * indicates significantly different from control cells (*p* < 0.05) and **^#^** significantly different from fucoxanthin-treated cells (*p* < 0.05); (**C**) with anti-Nrf2 and anti-phospho Nrf2. * and ** indicates significantly different from Nrf2 and phospho Nrf2 of control, respectively (*p* < 0.05), **^#^** and **^##^** indicates significantly different from Nrf2 and phosphoNrf2 of fucoxanthin-treated cells, respectively (*p* < 0.05); (**D**) with anti-GCLC and anti-GSSantibodies. * and ** indicates significantly different from GCLC and GSS of control, respectively (*p* <0.05), ^#^ and **^##^** indicates significantly different from GCLC and GSS of fucoxanthin-treated cells, respectively (*p* < 0.05).

To further elucidate the up-stream signaling pathway involved in fucoxanthin-mediated activation of Nrf2 and induction of GCLC and GSS, we examined activation of Akt, which is a signaling enzyme that is involved in the phosphorylation and nuclear translocation of Nrf2 [23]. Activation of Akt by fucoxanthin was assessed by performing Western blotting with an antibody against phosphorylated Akt. Fucoxanthin treatment increased phosphorylation of Akt (Figure 3A). A LY294002, phosphoinositide 3-kinase (PI3K)/Akt inhibitor, specifically represses the phosphorylation of Akt [24]. This inhibitor reduced the fucoxanthin-induced phospho Akt expression (Figure 3B). Furthermore, this inhibitor suppressed the fucoxanthin-induced Nrf2, GCLC and GSS expression (Figure 3C,D).

### 2.4. Fucoxanthin Promotes the Synthesis of GSH Catalyzed by GCLC and GSS

**Figure 4:**
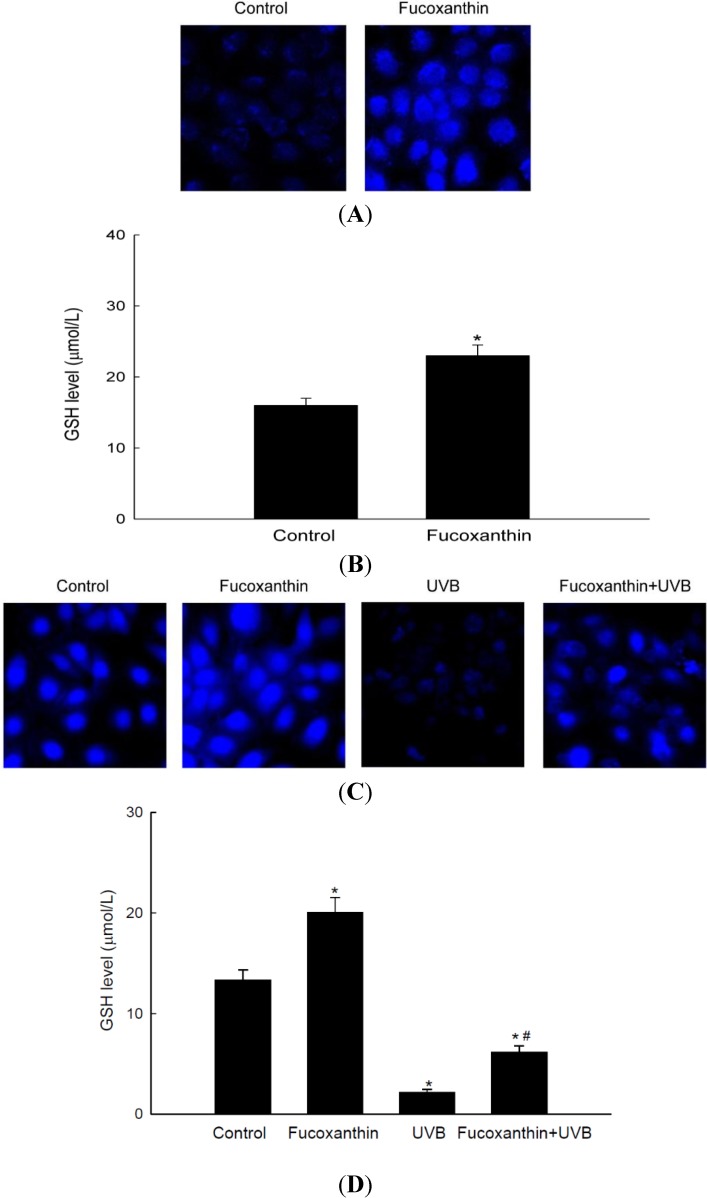
Effect of fucoxanthin on the level of reduced glutathione (GSH). The level of GSH was assessed in cells treated with 20 μM fucoxanthin for 12 h by (**A**) performing confocal microscopy after CMAC staining and (**B**) using a GSH detection kit. * indicates significantly different from control (*p* < 0.05). The level of GSH in UVB-treated HaCaT cells incubated for 12 h, with or without pretreatment with 20 μM fucoxanthin, was detected by (**C**) performing confocal microscopy after CMAC staining and (**D**) using a GSH detection kit. * indicates significantly different from control (*p* < 0.05) and **^#^** significantly different from UVB-irradiated cells (*p* < 0.05).

GSH is a tri-peptide formed via GCLC and GSS, and has powerful antioxidant effects against free radicals. GSH was detected by confocal microscopy using 7-amino-4-chloromethylcoumarin (CMAC), a dye that specifically labels GSH. The fluorescence intensity of CMAC, indicative of the level of GSH, was notably higher in fucoxanthin-treated cells than in control cells (Figure 4A). Consistently, fucoxanthin increased the concentration of GSH, as determined by a GSH detection kit (Figure 4B). To evaluate whether fucoxanthin induced GSH production to protect cells against ultraviolet B (UVB)-induced oxidative stress, cells were pretreated with fucoxanthin and then exposed to UVB irradiation. The level of GSH was reduced by UVB exposure, and this decrease was significantly restored in cells pretreated with fucoxanthin (Figure 4C,D). These data suggest that fucoxanthin partially recovers the reduction of GSH induced by UVB.

## 3. Materials and Methods

### 3.1. Materials

Anti-TATA-binding protein (TBP) and anti-phospho Nrf2 antibodies were purchased from Abcam, Inc. (Cambridge, MA, USA). Anti-Nrf2, anti-Akt, and anti-phospho Akt antibodies were purchased from Cell Signaling Technology (Beverly, MA, USA). Fucoxanthin, anti-GCLC and anti-GSS antibodies were purchased from Santa Cruz Biotechnology (Santa Cruz, CA, USA). [3-(4,5-Dimethylthiazol-2-yl)-2,5-diphenyltetrazolium] bromide (MTT) and an anti-β-actin antibody were purchased from Sigma-Aldrich Chemical Company (St. Louis, MO, USA). Cell Tracker™ Blue CMAC was purchased from Molecular Probes (Eugene, OR, USA). LY294002 was provided by Calbiochem (San Diego, CA, USA). All other chemicals and reagents were of analytical grade.

### 3.2. Cell Culture

The human keratinocyte cell line HaCaT was supplied by Amore Pacific Company (Gyeonggi-do, Korea) and maintained at 37 °C in an incubator with a humidified atmosphere of 5% CO_2_ and 95% air. Cells were grown in RPMI 1640 medium containing 10% fetal calf serum, streptomycin (100 μg/mL), and penicillin (100 units/mL).

### 3.3. Reverse Transcription-PCR (RT-PCR)

Total RNA was isolated from cells using the easy-BLUE™ total RNA extraction kit (iNtRON Biotechnology Inc., Seongnamsi, Korea). cDNA was amplified using 1 μL of reverse transcription reaction buffer, primers, dNTPs, and 0.5 U of Taq DNA polymerase in a final volume of 20 μL. The PCR conditions were initial denaturation at 94 °C for 5 min, followed by 26 cycles of 94 °C for 30 s, 63 °C for 45 s, and 72 °C for 1 min, and a final elongation step at 72 °C for 7 min. The following primers were used: human GCLC, forward (5′-AACCAAGCGCCATGCCGACC-3′) and reverse (5′-CCTCCTTCCGGCGTTTTCGC-3′); human GSS, forward (5′-GCCCCATTCACGCTCTTCCCC-3′) and reverse (5′-ATGCCCGGCCTGCTTAGCTC-3′); human GAPDH, forward (5′-TCAAGTGGGGCGATGCTGGC-3′) and reverse (5′-TGCCAGCCCCAGCGTCAAAG-3′). The amplified products were mixed with blue/orange 6 × loading dye, resolved by electrophoresis on a 1% agarose gel, stained with RedSafe™ nucleic acid staining solution (iNtRON Biotechnology Inc., Seongnamsi, Korea), and photographed under UV light using Image Quant™ TL analysis software (Amersham Biosciences, Uppsala, Sweden).

### 3.4. Western Blot Analysis

Cells were lysed on ice for 30 min in 100 μL lysis buffer (120 mM NaCl, 40 mM Tris (pH 8), and 0.1% NP-40) and centrifuged at 13,000× *g* for 15 min. Supernatants were collected and the protein concentrations were determined. Aliquots containing 40 μg of protein were boiled for 5 min and electrophoresed on 10% SDS-polyacrylamide gels. Proteins were transferred to nitrocellulose membranes, which were subsequently incubated with a primary antibody overnight at 4 °C. The membranes were further incubated with horseradish peroxidase-conjugated anti-immunoglobulin-G (Pierce, Rockford, IL, USA). Protein bands were detected using an enhanced chemiluminescence western blotting detection kit (Amersham Biosciences, Little Chalfont, Buckinghamshire, UK).

### 3.5. Immunocytochemistry

Cells at a density of 1.0 × 10^5^ cells/mL were seeded into a 4-well chamber slide. After incubation for about 16 h, cells were exposed to 20 μM fucoxanthin for a further 6 h. Subsequently, cells were fixed with 1% paraformaldehyde for 30 min and then washed three times with phosphate-buffered saline (PBS) for 5 min each time. Cells were permeabilized with PBS containing 1% Triton X-100 for 30 min and then washed with PBS. Cells were blocked with PBS containing 5% bovine serum albumin for 1 h at 37 °C, and then treated with an anti-Nrf2 antibody diluted in blocking medium (1:125 dilution) overnight. To visualize the primary anti-Nrf2 antibody, cells were treated with a FITC-conjugated secondary antibody (1:125) for 2 h. After washing with PBS, stained cells were mounted onto microscope slides in mounting medium containing DAPI (Vector, Burlingame, CA, USA). Images were collected using the LSM 510 program on a Zeiss confocal microscope.

### 3.6. ChIP Assay

Cells were processed using the SimpleChIP^®^ enzymatic chromatin IP kit (Cell signaling technology, Beverly, MA, USA) according to the manufacturer’s instructions. Briefly, proteins were cross-linked to DNA by adding 1% formaldehyde to the culture dishes and rocking on a platform for 10 min at room temperature. The cross-linking was stopped by the addition of glycine solution. Cells were washed twice with ice-cold PBS, pelleted by centrifugation, and re-suspended in 1 mL cell lysis buffer containing protease inhibitors. Soluble chromatin was sheared by sonication and then centrifuged at 15,000× *g*. Diluted supernatants were pre-cleared and blocked with protein A/G agarose, and the sonicated chromatin-DNA complex was precipitated overnight with the antibodies of interest. Bound DNA was eluted by incubating the beads in elution buffer, purified, and amplified using primers flanking the Nrf2-binding site within the promoters of the genes encoding human GCLC and GSS. The oligonucleotide containing the transcription factor-binding site of the GCLC and GSS promoter was obtained from Bioneer (Seoul, Korea). The ChIP procedure was analyzed by PCR using human GCLC and GSS promoter-specific primers as follows: GCLC, forward (5′-ATCTCCACGGTCCAGGTT-3′) and reverse (5′-CTCCCTCACCCTATCCATTT-3′); GSS, forward (5′-CTGGGAATAACCAGACACCTA-3′) and reverse (5′-CAGGTTCAAGCAATTCTCCTG-3′). The cycle parameters were as follows: initial denaturation at 95 °C for 5 min, followed by 40 cycles of 95 °C for 30 s, 60 °C for 30 s, and 72 °C for 30 s, and a final extension at 72 °C for 7 min. The amplified products were resolved by electrophoresis on a 3% agarose gel, stained with RedSafe™ nucleic acid staining solution, and photographed under UV light using Image Quant™ TL analysis software.

### 3.7. Luciferase Reporter Assay

HaCaT cells were transiently transfected with 0.5 μg of the luciferase reporter and 0.2 μg of the ARE expression vector using Lipofectamine™ 2000 (Invitrogen Corporation, Carlsbad, CA, USA). Co-transfection with 0.02 μg of pRL-TK Renilla reniformis luciferase served as a normalizing control. Luciferase assays were performed using the dual luciferase assay system (Promega, Madison, WI, USA).

### 3.8. Detection of GSH

For image analysis of GSH, cells were seeded in four-well chamber slides at a density of 1 × 10^5^ cells/mL. Sixteen hours after plating, cells were treated with 20 μM fucoxanthin and then irradiated with UVB 1 h later. After 12 h, 10 μM of CMAC was added to each well, and samples were incubated for an additional 30 min at 37 °C. After washing with PBS, the stained cells were mounted onto a chamber slide in mounting medium. Images were collected on a confocal microscope using the LSM 5 PASCAL software (Carl Zeiss, Jena, Germany). In addition, the GSH concentration was measured using a BIOXYTECH GSH-400 assay kit (Foster City, CA, USA).

### 3.9. Statistical Analysis

All measurements were performed in triplicate and all values are expressed as the mean ± of the standard error. The results were subjected to an analysis of variance followed by Tukey’s test to analyze differences between means. In each case, a *p* value less than 0.05 was considered statistically significant.

## 4. Conclusions

In this study, we demonstrated that fucoxanthin induced activation of nucleus Nrf2 via PI3K/Akt, which in turn activated the transcription of ARE-driven GCLC and GSS genes, leading the synthesis of antioxidant GSH. We recently reported that fucoxanthin did not show toxic effect on HaCaT cells at 20 μM and this concentration prevented cells against oxidative damage [21]. Therefore, we chose the same concentration to examine the effect of fucoxanthin on GSH induction in the present study.

GSH is an important biological antioxidant and has diverse functions in nutrient metabolism [25], gene expression, and DNA/protein synthesis [26], and particularly in eliminating oxidants [27]. GSH is formed from glutamate, cysteine, and glycine in a reaction that is catalyzed by two cytosolic enzymes, namely, GCLC and GSS [6]. The GSH is associated with the inhibition of tumor cell growth [28], prevention of apoptosis [29], and reduced inflammation [30]. In this study, fucoxanthin increased the mRNA and protein expression of GCLC and GSS (Figure 1). Nrf2, a major transcription factor of antioxidant enzymes, is tightly controlled by a master regulator Keap-1. Keap-1 has a high affinity for Nrf2 owing to its cysteine residues that form a covalent bond with Nrf2 [31]. Subsequently, Nrf2 signaling is switched off by Keap-1-mediated ubiquitination and degradation of Nrf2 [32]. However, the phospho form of Nrf2 can translocate into nucleus [33].

The nuclear Nrf2 recognizes the ARE sequence within the promoters of its target genes [34], and binding of Nrf2 to ARE sequences stimulates the transcription of genes that are involved in cellular defense. As shown in data, the phosphorylated Nrf2 expression was induced by fucoxanthin treatment (Figure 2A,B). The Nrf2 then binds to ARE sequence in GCLC and GSH promoters, which was assessed by ChIP assays (Figure 2C). The increased Nrf2 binding ability to ARE sequence in fucoxanthin-treated cells led to the increased transcriptional activity of Nrf2 (Figure 2D). These results indicated that fucoxanthin promotes release of Nrf2 from Keap-1 and subsequent translocation to nucleus, which induced the synthesis of GSH by enhancing expression of GSS and GCLC through interaction between Nrf2 and ARE sequence. It recently reported that fucoxanthin enhanced heme oxygenase-1 and NAD(P)H:quinone oxidoreductase 1 expression via activation of Nrf2/ARE system [35]. The phosphorylation of Nrf2 plays a pivotal role in its nuclear accumulation, and this phosphorylation can occur via Akt pathways [36]. Akt is a classic signal-transducing protein which can activate the primary cellular defense mechanism Nrf2/ARE in skin cells [37]. In our study, fucoxanthin treatment increased the level of phosphorylated Akt, which is the active form of this kinase (Figure 3A) and could elevate the nuclear level of Nrf2 (Figure 2A). Furthermore, LY294002, a specific inhibitor of PI3K/Akt, significantly suppressed the active form of Akt (Figure 3A) which resulted in reduction of Nrf2 accumulation, following decreased protein expression of GSS and GCLC (Figure 3B–D). In addition, working in concert with the effects of fucoxanthin on GSS and GCLC expression, the amount of GSH was increased in fucoxanthin-treated cells, as indicated by the increased fluorescence intensity of CMAC (Figure 4A) and the increased concentration of GSH (Figure 4B). In our system, UVB irradiation, an inducer of oxidative stress, suppressed the GSH level. However, treatment with fucoxanthin prior to UVB damage partially mitigated the reduction in GSH levels (Figure 4C,D).

In conclusion, fucoxanthin substantially increased the mRNA and proteins levels of GCLC and GSS in human keratinocytes, and these effects were dependent on the nuclear translocation of Nrf2 following its phosphorylation by the protein kinase Akt. In addition, this study demonstrates that the Akt/Nrf2 pathway plays an essential role in the mechanism underlying the effects of fucoxanthin. Taken together, one of the major ways by which fucoxanthin treatment prevents or eliminates oxidative damage is to enhance the Akt/Nrf2/GSH-dependent antioxidant response.
